# Peanut Skin Color: A Biomarker for Total Polyphenolic Content and Antioxidative Capacities of Peanut Cultivars

**DOI:** 10.3390/ijms10114941

**Published:** 2009-11-11

**Authors:** Yvonne Chukwumah, Lloyd T. Walker, Martha Verghese

**Affiliations:** Department of Food and Animal Sciences, Alabama A&M University, P O Box 1628, Normal, AL 35762, USA; E-Mails: yvonne.chukwumah1@aamu.edu (Y.C.); martha.verghese@aamu.edu (M.V.)

**Keywords:** peanut (*Arachis hypogaea* L.), groundnut, skin color, polyphenols, flavonoids proanthocyanidin, cultivars, biomarker

## Abstract

Attempts to establish a relationship between peanut skin color (PSC) and total flavonoid (TF) content have produced inconclusive results. This study investigated the potential of PSC as a biomarker for polyphenol content and antioxidant capacity. Peanut cultivars were objectively evaluated for their skin color, total phenolic (TP), flavonoid (TF), proanthocyanidin (TPC) contents and antioxidant capacities (AC). Their relationship was determined by Pearson’s correlation analyses. TP had stronger correlations with CIE a*, hue angle and AC (r^2^ = 0.77, 0.82 and 0.80, respectively) compared to TF. Therefore, hue angle of peanut skin may be used as a biomarker for TP content rather than TF.

## 1. Introduction

Oxidative stress has been identified as a key factor in the development of chronic diseases such as cancer and cardiovascular diseases which, according to the CDC Report on National Vital Statistics for 2006, are still the leading causes of death in the United States [1]. The development of these diseases as well as their metabolic risk factors (hyperlipidemia, hypertension, diabetes and obesity) has been attributed to several factors which are based primarily on diet and lifestyle [2]. Key recommendations for the prevention and mitigation of these conditions have been centered on increased consumption of plant-based foods (fruits, vegetables and nuts) that are rich in phenolic compounds [3]. Polyphenols play a role in the prevention of degenerative diseases, particularly cardiovascular diseases and cancers through the modulation of oxidative stress mediated through their antioxidative properties [4]. These biologically active compounds have been reported to have anti-inflammatory [5–7], anti-carcinogenic [8–10], and anti-ischemic properties [11].

Peanut is a dietary source of biologically active polyphenols such as the stilbene *trans*-resveratrol [12], flavonoids such as the proanthocyanidins [13], flavonols such as quercetin [14] and the isoflavones daidzein and genistein. Several studies have shown that the amounts of these plant metabolites can vary as a result of variations in plant cultivar, maturity, color, size and growth conditions. Peanut skin has been reported to be rich in polyphenols and there have been several unsuccessful attempts by legume breeders to establish a relationship between seed coat color and total flavonoid content. Todd and Vodkin, [15] and Wang *et al.* [14] studied the relationship between legume seed coat and total flavonoid content. Results of these studies have remained controversial as their findings contradict each other. Peanut skin color varies from light brown to deep red and most color pigments in plants especially red, purple and blue belong to the flavonoid class of anthocyanins, with other flavonoid compounds acting as co-pigments.

Peanuts in the US are classified into four types, the Runner and Virginia market types belonging to the subspecie *hypogaea* and the Spanish and Valencia market-types belonging to the subspecie *fastigiata*. According to the American Peanut Council, most of the peanuts cultivated and consumed widely in the U.S. are the Runner market-type which accounts for approximately 80% of the total peanut produced in the U.S. while Valencia cultivars are the least cultivated and account for approximately 1% of total peanut produced. Valencia peanuts, which are cultivated predominantly in New Mexico, are processed and consumed as in-shell snacks [16,17]. Studies on the phytochemical composition and antioxidant capacities of peanuts have been on cultivars of market-types other than Valencia. This may be due to its low cultivation and consumption level. If recommendations on consumption levels of phytochemical rich foods such as peanut are to be made for the prevention of chronic diseases, information on representative population of each food must be taken into account.

Thus, the objectives of our study are to objectively evaluate peanut skin color of cultivars of various market-types and to establish its relationship with the phytochemical content and antioxidant capacities of the whole seed. Although the phytochemicals are concentrated in the skin, which makes up about 4% by weight of the whole kernel, there are still some contributions, though minor, from the kernel and thus the whole seed (skin and kernel) will be evaluated for phytochemical content. Our goal is to determine the potential of the peanut skin color as an indicator of the phenolic/flavonoid content of peanut seeds.

## 2. Results and Discussion

### 2.1. Peanut Skin Color

**Figure 1.**
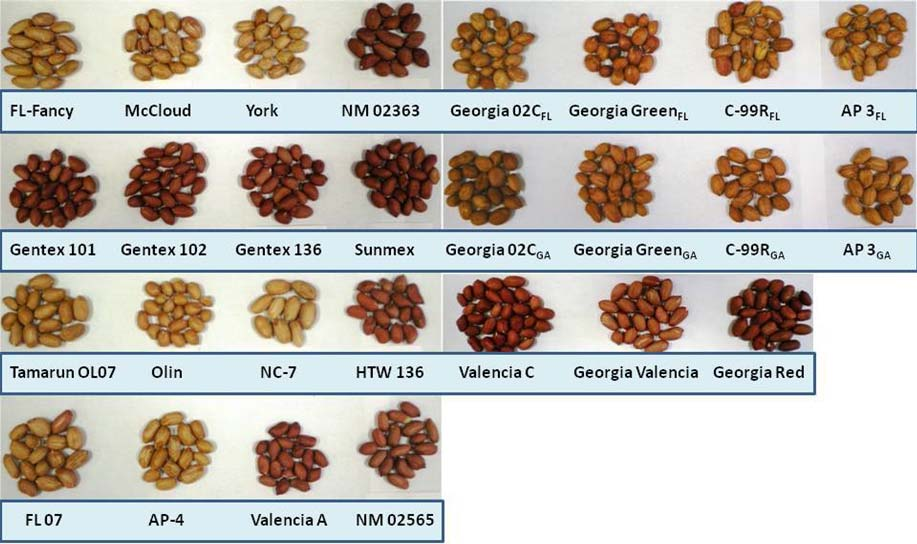
Peanut cultivars from the four peanut market-types cultivated in the US.

Peanut skins have colors ranging from light brown to deep red. The mean L* (lightness) and b* (yellowness) value for all cultivars were 43.8 and 19.3, respectively (Table 1). All cultivars studied with the exception of Valencia cultivars and the Runner cultivar, Georgia green from Florida, had L* and b* values that were significantly (p < 0.05) higher than the overall mean, indicating the darkness of their skin color (Figure 1). However, Valencia cultivars have significantly higher CEI a* (redness) and hue values compared to cultivars of other market types with the exception of the Runner cultivars, FL-07 and Georgia green from Florida, which have similar degree of redness as NM 02565 (Table 1). Higher redness and hue values indicate that these cultivars are highly pigmented and therefore could possibly be used as a marker for selection of polyphenol-rich cultivars should there be a very strong positive correlation between color and phenolic content.

### 2.2. Total Flavonoids

**Figure 2.**
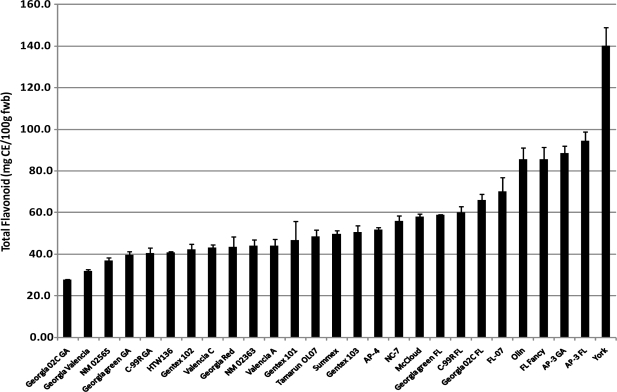
Total flavonoid content of peanut cultivars from four peanut market-types.

The flavonoid content of cultivars from the four peanut market-types is shown in Figure 2. The amounts range from 27.6–139.9 mg CE/100 g fresh peanut, with an average flavonoid content of 56.4 mg CE/100 g. The distribution of flavonoid content among cultivars investigated is shown in Figure 2 in which 16 of the 27 cultivars had flavonoid contents between 40.0–60.0 mg CE/100 g. Of these 16 cultivars, nine were Valencia market-type. Flavonoids are responsible for imparting color in various plant parts. Todd and Vodkin reported a corresponding high content in color imparting flavonoids in soybean seed coat [15]. Objective measurements of color intensity (CEI values) of peanut skin showed a wide range of L* and a* values (Table 1). The relationship between legume seed coat color and total flavonoid content, although very important to legume breeder, has been controversial as the relationship reported by Todd and Vodkin was not observed in the study by Wang *et al.* [14]. They investigated accessions of legumes including peanuts and soybeans but found no relationship between seed coat color and flavonoid content. They explained that the lack of correlation was due to genetic differences as samples were from different continents. Similar findings were observed in our study as there were poor correlations between CIE L*, a*, b* and hue values and total flavonoid content with correlation coefficients (r^2^) of 0.57, −0.46, 0.57 and −0.54, respectively (Table 2). Cultivars with higher total flavonoid content, mainly Runner cultivars cultivated in Florida, had significantly lower CIE a* values. This means that although a cultivar may be high in total flavonoid, those that contribute to the skin pigments may not necessarily be in high concentrations. However, a much stronger positive relationship (r^2^ = 0.90) was observed between total flavonoids and proanthocyanidin content (Table 2) indicating that proanthocyanidins are the major flavonoids present in peanut skin.

### 2.3. Total Phenolics

**Figure 3.**
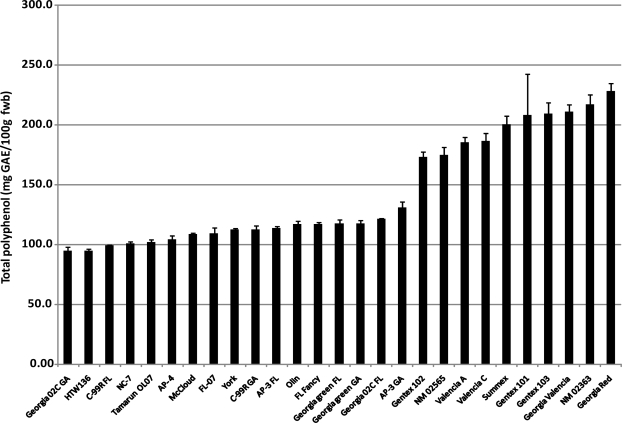
Total polyphenol content of peanut cultivars from four peanut market-types.

The total polyphenol content of the peanut cultivars investigated in this study ranged from 94.4–228.4 mg GAE/100 g fresh peanut with an average content of 143.5 mg GAE/g (Figure 3). The distribution of the total phenolic content among the 27 cultivars is shown in Figure 3. Twelve of the 27 cultivars have total phenolic contents between 100–120 mg GAE/100 g. These cultivars, with the exception of Olin, are all Runner market-type cultivars. On the other hand, Valencia cultivars with the exception of HTW136 had significantly higher total phenolic content ranging from 173.1–228.4 mg GAE/100 g peanut. Although the total phenolic content of peanut has been reported in various studies by previous authors, these data were on Runner varieties and would therefore represent the total polyphenol content in approximately 50% of peanut consumed in the US [17]. Correlation analysis of peanut skin color and total polyphenols shows moderate positive correlation for CIE a* and hue values (0.77 and 0.82, respectively) (Table 2). This suggests that the hue angle of peanut skin may be indicative of the total polyphenol content rather than its flavonoid content.

### 2.4. Total Proanthocyanidins

**Figure 4.**
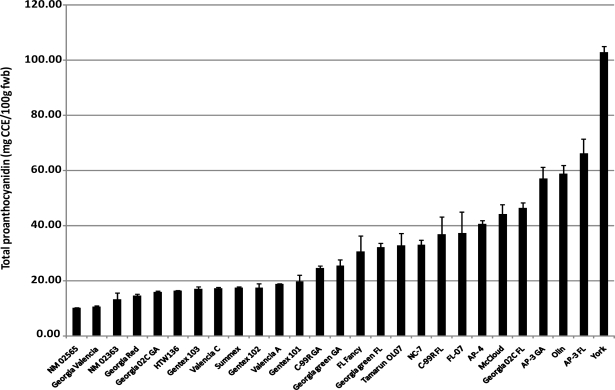
Total proanthocyanidin content of peanut cultivars from four peanut market-types.

Peanuts have been shown to contain the proanthocyanidins catechin and epicatechin in the monomeric, dimeric, trimeric and tetrameric forms, most of which are located in the peanut skin [18]. Results obtained in this study show that there is a wide variation in the total proanthocyanidin content of whole peanuts among the 27 cultivars investigated. Their proanthocyanidin content expressed as cyanidin chloride equivalent (CCE) range from 10.1–103.0 mg CCE/100 g fresh peanut with an average of 31.7 mg CCE/100 g (Figure 4). Frequency distribution of these values shows that most of the cultivars in this study (63%, 17 cultivars) have proanthocyanidin contents below the mean value and these include all the Valencia market-type cultivars and some Runner market-types (Figure 4). Gu *et al*. [13] in their study of proanthocyanidin content of foods in American diet estimated the amount of proanthocyanidin in roasted peanut to be 15.6 mg/100 g fresh weight. In this study, Georgia 02C cultivated in the state of Georgia had a value similar to the reported value. Most of the cultivars investigated had higher values ranging from 15.7–44.1 mg CCE/100 g (Figure 4) comprising Valencia, Virginia and Runner market-types with Valencia cultivars having the least values compared to the other market-types. Correlation analysis showed moderate to weak correlations between peanut skin color and total proanthocyanidin content, especially CEI L* and b* values (0.70 and 0.74, respectively) and therefore cannot be selected-for using peanut color.

### 2.5. Trolox® Equivalent Antioxidant Capacity (TEAC)

**Figure 5.**
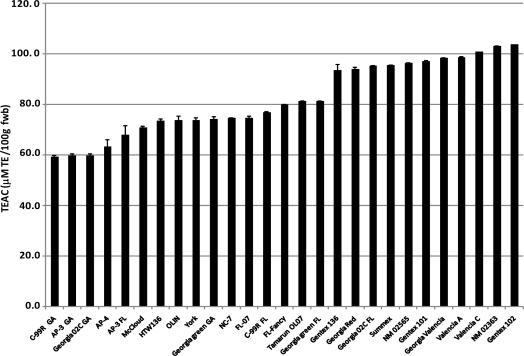
Trolox equivalent antioxidant capacities (TEAC) of peanut cultivars from four peanut market-types.

Natural polyphenols are potent antioxidants and therefore plant-based foods that are rich in polyphenols have an inherent ability to exhibit antioxidative properties [19]. The total antioxidant capacities ranged from 59.1–103.8 mM TE/100 g, with a mean value of 82.3 mM TE/100 g (Figure 5). Valencia cultivars, with the exception of HTW136, had significantly (p < 0.05) higher antioxidant capacities than cultivars of other market types, with the exception of Georgia 02C from Florida. The distribution of the antioxidant capacities shows that approximately 70% (19 cultivars) have antioxidant capacities between 70–100 mM TE/100 g. Correlation analysis showed significant (p < 0.05) correlation between peanut skin color and antioxidant capacity however, the strength of the relationship was moderate for CIE a* and hue angle (Table 2) and cannot therefore be estimated by the skin color. However, there was a stronger correlation between antioxidant capacity and total polyphenol content (r^2^ 0.80). This indicates a positive relationship between TP content and antioxidant capacity but may not be strong enough to infer that peanuts high in total polyphenolic content have high antioxidant capacity.

## 3. Experimental Section

### 3.1. Materials

Twenty seven peanut cultivars (14 Runner, 11 Valencia, one Virginia and one Spanish) were obtained from University of Florida, University of Georgia (UGA), New Mexico State University and Texas A & M University. Their colors were measured objectively with a HunterLab colorimeter and the peanuts were stored at 4 °C until analyzed. The cultivars from UGA were the Runner market-type (AP-3, C-99R, Georgia Green and Georgia 02C) while cultivars from Texas comprised Spanish, Virginia, Valencia and Runner market-types (Olin, NC-7, HTW136 and Tamarun OL07). Nine Runner cultivars were obtained from Florida (AP-3, AP-4, C-99R, Georgia Green, Georgia 02C, FL-07, FL Fancy, McCloud and York) and 10 Valencia cultivars from New Mexico (Valencia A, Valencia C, Gentex 101, Gentex 102, Gentex 136, Sunmex, Georgia Red, Georgia Valencia, NM 02363, and NM 02565). The number and ratio of cultivars used in the study was largely dependent on availability. The large number of Valencia cultivars used in this study was considered necessary since there are no known phytochemical studies on Valencia cultivars and previous studies in our laboratory (data not included) have shown a similarity between the phytochemical profiles of Runner, Virginia and Spanish cultivars. Pure standards of gallic acid, Trolox^®^ and catechin were purchased from Sigma Chemical Company (St. Louis, MO, USA), cyanidin chloride was purchased from Alexis Biochemicals (San Diego, CA, USA).

### 3.2. CEI Color Measurements

CEI L*, a* and b* values were measured using a HunterLab colorimeter (Colorflex Model 45/0, HunterLab Inc., Reston VA, USA) that was calibrated using a white reference standard tile. Whole peanuts with their skin intact were evenly spread on the bottom of the colorimeter cup. Three sets of readings were obtained per sample by rotating the cup a third of a turn each time. The L*, a*, and b* values corresponds to lightness/darkness, redness/greenness, and yellowness/blueness, respectively. The hue angle, an attribute of color perception, is a measure that distinguishes red from green and blue from yellow was calculated from a* and b* values using the formula: hue angle = tan^–1^ b*/a*.

### 3.3. Preparation of Sample Extracts

Peanut samples were milled and defatted in 10 vol of hexane at room temperature. The defatted samples were used for extraction of polyphenols. Four grams of sample was extracted in 6 vol of 80% aqueous methanol. The suspension was sonicated (Bransonic IC, Model IC1216-25-12, Branson Corporation, Danbury, CT, USA) at 25 kHz for 20 min at ambient temperature and then centrifuged at 2,100 g for 20 min at 4 °C. The supernatant was collected and concentrated under reduced pressure using a rotary evaporator at 38 °C. The extract was then analyzed for phenolic, flavonoid and proanthocyanidin content. The extracts were also evaluated for antioxidant capacities.

### 3.4. Total Flavonoids

Total flavonoid content of the peanut extracts was determined as described by Jia *et al.* [20]. Briefly, 150 μL of 5% sodium nitrite was added to 2 mL of appropriately diluted sample extract. After 5 min, 150 μL of 10% aluminum chloride was added. One milliliter of 1 M sodium hydroxide and 1.2 mL of distilled water were added to the mixture after 10 min. The absorbance was read at 510 nm using a spectrophotometer (Genesys 6, Thermoelectron Corporation, Madison, WI, USA). A catechin standard curve (5 – 25 μg/mL) was established and used to calculate the flavonoid content of the sample extracts and these were expressed as catechin equivalent (mg CE/100 g) on fresh weight basis.

### 3.5. Total Phenolics

Total polyphenol content of the peanut extracts was determined using the method described by Singleton and Rossi [21]. Folin-Ciocalteau reagent (1.25 mL of 0.2 N Folin Ciocalteau) was added to 250 μL of appropriately diluted sample extracts. After 8 min, 1 mL of 7% sodium carbonate was added. The mixture was kept in a water bath at 40 °C for 30 min after which 10 mL of distilled water was added and vortexed. The absorbance was read at 750 nm. A gallic acid standard (100–500 μg/mL) curve was established and used to calculate the total polyphenol content of the sample extracts which were expressed as gallic acid equivalent (mg GAE/100 g) on fresh weight basis.

### 3.6. Total Proanthocyanidins

Total proanthocyanidin content of the peanut extracts was determined by acid-butanol assay adapted from Porter *et al.* [22]. Briefly, 0.25 mL of the peanut extract placed in a boiling tube and 3 mL of *n*-butanol–HCl solution (95:5 v/v) and 0.1 mL of NH_4_Fe(SO_4_)_2_ · 12H_2_O in 2 M HCl were added. The tubes were capped and incubated for 40 min at 95°C. After cooling, the absorbance was read at 550 nm. A standard curve using cyanidin chloride (100–500 μg/mL) was established and used to calculate the amount of proanthocyanidin in the sample extracts. Results were expressed as cyanidin chloride equivalent (mg CCE/100 g) on a fresh weight basis.

### 3.7. Trolox Equivalent Antioxidant Capacity (TEAC Assay)

The ABTS/AAPH method [23] was used to evaluate the ability of the peanut sample extracts to scavenge preformed radical cation ABTS+. The radical ion was generated by a reaction between ABTS (2.5 mM) and activated AAPH (2 mM) in phosphate buffer (0.1 M) pH 7.4 with an absorbance of 0.70 ± 0.1 at 734 nm. To 0.99 mL of the ABTS+ solution, 0.01 mL of sample extract was added and the decay in absorbance was followed for 6 min at 734 nm. A blank measurement was recorded for each sample. A standard curve using Trolox^®^ (3–12 mM) was established and used to calculate the antioxidant capacity. TEAC values were expressed in mM Trolox^®^ equivalent (TE)/100 g fresh weight sample.

### 3.8. Statistical Analysis

The experiment was replicated three times. Statistical analysis (ANOVA) of data were done using the SAS version 9.1 software, and where significant, mean separations were obtained using Tukey’s Studentized test at p ≤ 0.05. The relationships between peanut skin color, phenolic, flavonoid contents and antioxidant capacity were determined statistically by Pearson’s correlation analysis.

## 4. Conclusions

The redness and hue angle of the peanut skin had strong correlations with total polyphenol content and not the total flavonoid content. They also had good correlations with antioxidant capacity supporting the strong correlation between total polyphenols and antioxidant capacity suggesting that the hue angle of the peanut skin may be used as a biomarker of total polyphenol content. On the other hand, total flavonoid content had a stronger correlation with total proanthocyandins and therefore may be used as an indicator for proanthocyanidin content of peanuts cultivars.

## Figures and Tables

**Table 1.** Peanut skin color (CIE L*, a*, b*) and hue angle of 27 peanut cultivars.

**Peanut Cultivar**	**State**^W^	**Market type**^x^	**CIE Color**^y^			
			L*	a*	b*	**Hue angle**
AP-3	FL	R	49.34 ± 0.14^h^	14.24 ± 0.20^j^	23.84 ± 0.20^bc^	0.54 ± 0.01^k^
AP-4	FL	R	47.74 ± 0.08^i^	15.97 ± 0.09^fghi^	22.21 ± 0.25^de^	0.62 ± 0.01^hij^
C-99R	FL	R	50.32 ± 0.04^g^	12.97 ± 0.03^k^	21.54 ± 0.06^ef^	0.54 ± 0.00^k^
Georgia Green	FL	R	43.53 ± 0.09^k^	19.29 ± 0.17^d^	18.42 ± 0.20^g^	0.81 ± 0.01^f^
Georgia 02C	FL	R	50.46 ± 0.06^fg^	15.21 ± 0.06^hij^	22.92 ± 0.05^cd^	0.59 ± 0.00^ijk^
FL-07	FL	R	45.92 ± 0.20^j^	19.43 ± 0.09^d^	22.03 ± 0.17^de^	0.72 ± 0.01^g^
FL Fancy	FL	R	51.01 ± 0.19^ef^	14.37 ± 0.09^j^	21.72 ± 0.14^ef^	0.58 ± 0.01^ijk^
McCloud	FL	R	53.21 ± 0.12^c^	15.45 ± 0.21^hi^	24.04 ± 0.28^b^	0.57 ± 0.01^k^
York	FL	R	51.08 ± 0.08^e^	14.99 ± 0.13^ij^	23.82 ± 0.15^bc^	0.56 ± 0.01^k^
Olin	TX	S	51.81 ± 0.16^d^	17.37 ± 0.07^e^	23.72 ± 0.17^bc^	0.63 ± 0.01^hi^
NC-7	TX	Vir	54.63 ± 0.42^a^	13.19 ± 0.64^k^	21.34 ± 0.62^ef^	0.55 ± 0.03^k^
HTW136	TX	Val	37.97 ± 0.31^n^	21.69 ± 0.62^bc^	14.06 ± 0.60^jk^	1.00 ± 0.03^cd^
Tamarun OL07	TX	R	53.87 ± 0.14^b^	16.07 ± 0.85^fgh^	25.67 ± 0.06^a^	0.56 ± 0.00^k^
AP-3	GA	R	48.75 ± 0.12^h^	15.88 ± 0.16^ghi^	24.56 ± 0.15^b^	0.57 ± 0.01^jk^
C-99R	GA	R	43.65 ± 0.16^k^	16.58 ± 0.19^efg^	22.01 ± 0.22^de^	0.65 ± 0.01^h^
Georgia Green	GA	R	47.05 ± 0.03^i^	16.92 ± 0.19^ef^	21.74 ± 0.17^e^	0.66 ± 0.01^h^
Georgia 02C	GA	R	40.10 ± 0.30^l^	16.54 ± 0.45^efg^	20.70 ± 0.53^f^	0.67 ± 0.03^gh^
Valencia A	NM	Val	35.43 ± 0.18^q^	24.62 ± 0.43^a^	14.79 ± 0.29^hij^	1.03 ± 0.02^abc^
Valencia C	NM	Val	36.12 ± 0.18^op^	24.96 ± 0.38^a^	14.37 ± 0.33^ijk^	1.05 ± 0.02^ab^
Gentex 101	NM	Val	33.79 ± 0.07^r^	24.15 ± 0.06^a^	13.83 ± 0.14^jk^	1.05 ± 0.01^a^
Gentex 102	NM	Val	35.72 ± 0.25^pq^	24.14 ± 0.06^a^	15.58 ± 0.16^h^	1.00 ± 0.01^bcd^
Gentex 136	NM	Val	37.52 ± 0.32^n^	24.67 ± 0.71^a^	15.38 ± 0.61^hi^	1.01 ± 0.03^abc^
Sunmex	NM	Val	36.45 ± 0.22^o^	22.60 ± 0.52^b^	14.77 ± 0.51^hij^	0.99 ± 0.03^cd^
Georgia Red	NM	Val	32.71 ± 0.08^s^	22.28 ± 0.19^b^	13.58 ± 0.02^k^	1.02 ± 0.01^abc^
Georgia Val	NM	Val	38.91 ± 0.04^m^	20.69 ± 0.14^c^	14.74 ± 0.27^hij^	0.95 ± 0.01^de^
NM 02363	NM	Val	35.81 ± 0.29^pq^	20.77 ± 0.58^c^	13.89 ± 0.60^jk^	0.98 ± 0.03^cd^
NM 02565	NM	Val	39.49 ± 0.02^m^	18.75 ± 0.03^d^	14.84 ± 0.07^hij^	0.90 ± 0.00^e^
Mean ± SEM			43.81 ± 0.78	18.66 ± 0.43	19.26 ± 0.47	0.77 ± 0.02

W State cultivated;

X market type (R –Runner, S-Spanish, Val-Valencia, Vir-Virginia).

y Values are means ± SD of three replicates. Mean separation by Tukey’s studentized test at p ≤ 0.05.

a-s Means with the same letter in a column are not significantly (p < 0.05) different.

**Table 2.** Pearson’s correlation of peanut skin color, total polyphenols, flavonoids, proanthocyanidins and antioxidant capacity (TEAC).

	**Pearson’s Correlation coefficient (r^2^)**			
	L*	a*	b*	**Hue**
	
**Total Polyphenols**	−0.78	0.77	−0.80	0.82
**Total Flavonoids**	0.57	−0.46	0.57	−0.54
**Total Proanthocyanidins**	0.70	−0.60	0.74	−0.70
**TEAC**	−0.62	0.70	−0.75	0.75
	**TP**	**TF**	**TPC**	**TEAC**

**Total Polyphenols**	1.00	−0.35	−0.52	0.80
**Total Flavonoids**		1.00	0.90	−0.33^Z^
**Total Proanthocyanidins**			1.00	−0.51
**TEAC**				1.00

n = 81;

Z Correlation coefficient is not significant at p ≤ 0.05.
